# Integration, heterochrony, and adaptation in pedal digits of syndactylous marsupials

**DOI:** 10.1186/1471-2148-8-160

**Published:** 2008-05-25

**Authors:** Vera Weisbecker, Maria Nilsson

**Affiliations:** 1 School of Biological, Earth and Environmental Sciences, University of New South Wales, UNSW/Sydney, NSW 2052, Australia; 2 Lund University, Department of Cell and Organism Biology, Genetics, Division of Evolutionary Molecular Systematics, Sölvegatan 29, S-223 62 Lund, Sweden

## Abstract

**Background:**

Marsupial syndactyly is a curious morphology of the foot found in all species of diprotodontian and peramelemorph marsupials. It is traditionally defined as a condition in which digits II and III of the foot are bound by skin and are reduced. Past treatments of marsupial syndactyly have not considered the implications of this unique morphology for broader issues of digit development and evolution, and the ongoing debate regarding its phylogenetic meaning lacks a broad empirical basis. This study undertakes the first interdisciplinary characterisation of syndactyly, using variance/covariance matrix comparisons of morphometric measurements, locomotor indices, ossification sequences, and re-assessment of the largely anecdotal data on the phylogenetic distribution of tarsal/metatarsal articulations and "incipient syndactyly".

**Results:**

Syndactylous digits have virtually identical variance/covariance matrices and display heterochronic ossification timing with respect to digits IV/V. However, this does not impact on overall locomotor adaptation patterns in the syndactylous foot as determined by analysis of locomotor predictor ratios. Reports of incipient syndactyly in some marsupial clades could not be confirmed; contrary to previous claims, syndactyly does not appear to impact on tarsal bone arrangement.

**Conclusion:**

The results suggest that marsupial syndactyly originates from a constraint that is rooted in early digit ontogeny and results in evolution of the syndactylous digits as a highly integrated unit. Although convergent evolution appears likely, syndactyly in Diprotodontia and Peramelemorpha may occur through homologous developmental processes. We argue that the term "syndactyly" is a misnomer because the marsupial condition only superficially resembles its name-giving human soft-tissue syndactyly.

## Background

**Figure 1:**
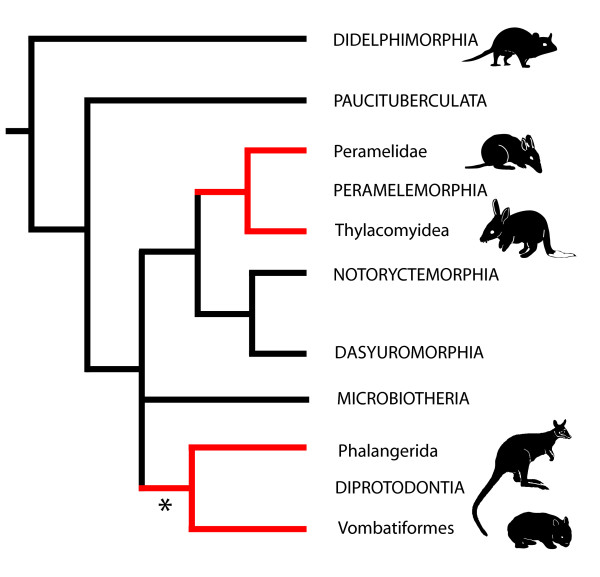
**Phylogeny of marsupial orders**. Figure based on a consensus of Amrine-Madsen et al. (2003) and Nilsson et al. (2004), with unresolved position of Microbiotheria. Red branches denote clades where syndactyly occurs.

The occurrence of homoplasy is problematic in phylogenetic reconstruction and the tracing of morphological evolution [1,2]. This is particularly the case when the evolution of a homoplastic trait is perceived as relatively complex and therefore deemed unlikely to have evolved more than once. A classic example that has sustained nearly two centuries of controversial and often heated debate is the character of marsupial syndactyly. This is commonly defined as a peculiar phenotype in which digits II and III of the marsupial foot are tightly connected by a common sheath of skin at least to the base of the intermediate phalanx [3,4]. The trait occurs in all species of two Australasian marsupial orders (Fig. 1), Peramelemorpha (bilbies and bandicoots; 21 species) and Diprotodontia (koalas, wombats, possums, kangaroos, and allies; approx. 141 species). Together, these two clades represent nearly 50% of marsupial species [5], Diprotodontia being the most ecologically and locomotorily diverse extant marsupial clade [6,7].

Marsupial syndactyly was one of the first characters used for marsupial classification, [8-12]. It has been reasoned that a character as complex as syndactyly could have only evolved once within marsupials [8,12-16], and peramelemorphs and diprotodontians were commonly grouped in the clade of Syndactyla [11,16,17]. Famously, this was at odds with the division of marsupials based on dentition into "diprotodont" (including Diprotodontia, and sometimes Paucituberculata, which possess two large procumbent incisors) and "polyprotodont" clades [peramelemorphs and all other marsupial clades [6,10,18]]. However, with the advent of molecular systematics, it became widely accepted [with some exceptions; [16,19]] that Diprotodontia and Peramelemorpha are not sister groups [20-23]. As Figure 1 shows, this arrangement favours homoplastic origins of syndactyly in Peramelemorpha and Diprotodontia [18,21,23-25]. However, there have also been suggestions that syndactyly may be plesiomorphic for larger marsupial clades, and has subsequently been lost in some clades [23,26,27].

**Figure 2:**
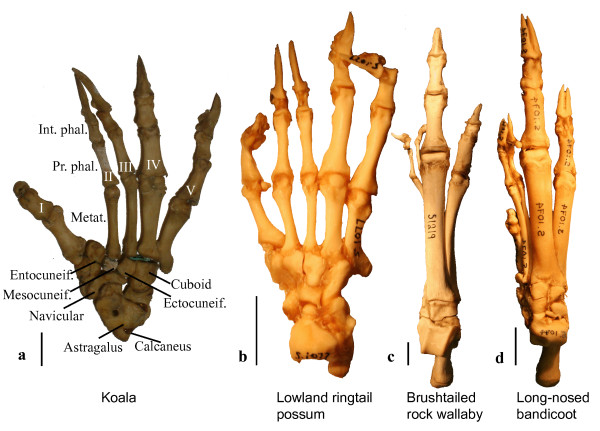
**Syndactylous feet**. Explanation of terminology and images of the right pedal skeleton of four syndactylous marsupials, showing tarsal bones and separated digits of a koala (a), a lowland ringtail possum *Pseudochirulus canescens *(b), a brush-tailed rock wallaby (c), and a long-nosed bandicoot *Perameles nasuta *(d). Note that a-c are diprotodontian marsupials, while d is a peramelemorph. Int. phal, intermediate phalanx; Metat., metatarsal; Prox. Phal, proximal phalanx. Roman numerals refer do digit numbers. Scale bar = 1 cm.

As a matter of course, it has been assumed that marsupial syndactyly arose under selective pressure, but what the functional relevance of syndactyly is has never been convincingly argued. A number of researchers suggested that the comparatively small size ("reduction") of syndactylous digits in most species is related to arboreal locomotion in putatively arboreal ancestors of the syndactylous clades [13,28,29]. This scenario has been contested because arboreal Diprotodontia have particularly strongly developed syndactylous digits [3,30]. A second hypothesis suggests that the function of syndactylous digits as "grooming digits" confers a selective advantage [3,11,18,31]. However, grooming organs are common among placental mammals without comparable extensive transformations in pedal morphology [3,13]. As such, the adaptive implications of marsupial syndactyly remain elusive. However, it has never been considered that it is a unique phenomenon with implications transcending its local importance. No extant tetrapod clade displays a trait which, without a clear-cut locomotor function, impacts as heavily and consistently on autopodial morphology of an ecologically diverse order. Such exceptions have the potential to inform our understanding of evolutionary and developmental patterns of tetrapod autopodial evolution [32], but little empirical information exists on syndactyly, which stems mainly from a few older dissection-based studies [3,33-35]. As such, syndactyly displays some intriguing but largely unexplored characteristics. For example, syndactyly is not an exclusively soft-tissue related condition but majorly affects the digits (Fig. 2). The characteristic morphology of digits II and III suggests that they evolve as a distinct unit within the syndactylous foot [4], but this has never been tested. The result of the unusual anatomy of syndactylous digits is that digit III never forms the main axis of the foot in syndactylous marsupials [36], which is rare among tetrapods [37,38]. Instead, the marginal digits IV and V are extensively developed, while digits II and III are considered "reduced" [13,31]. Nevertheless, locomotion in syndactylous marsupials (particularly Diprotodontia) is more diverse than in all other marsupials and many placental orders, catering for plantigrade terrestrial walking and bounding, grasping arboreal locomotion, and the unique hopping gait of kangaroos; this is also reflected in pedal diversity (Fig. 2). However, patterns of functional adaptation in the syndactylous foot and particularly of digits II and III remain to be explored. Marsupial syndactyly is also apparent very early in pedal development, which has lead to the as yet untested suggestion of heterochronic change of foot development in syndactylous species [13]. This is an interesting suggestion because heterochronic change in early ontogeny can help to explain evolutionary transformations of morphological traits [39-41]. Lastly, contextualisation of syndactyly in an evolutionary framework is to date impossible because the evidence regarding its distribution has not been comprehensively assessed; two lines of evidence have been deemed important in this respect. A range of didelphid species have been termed "incipiently syndactylous" (Table 1). This has been considered evidence that syndactyly may be more plesiomorphic within marsupials than has been generally recognized [23,26,42], although the existence of incipient syndactyly has been contested [30,42,43]. Contrary to this hypothesis, it was suggested that tarsal/metatarsal articulation patterns in peramelemorphs, which differ from the plesiomorphic mammalian condition, are a consequence of convergent acquisition of syndactyly [8,15].

**Table 1:** List of species considered incipiently syndactylous

Species	Author
*Caluromys derbianus*	Kirsch 1977, Bensley 1903
Genus *Marmosa*	Bensley 1903, Tate 1933, Kirsch 1977
*Monodelphis orinoci*	Kirsch 1977
*Thylamys pusilla*	Bensley 1903
*Gracilianus microtarsus*	Bensley 1903
*Philander opossum*	Bensley 1901
*Chironectes minimus*	Hall 1987
*Notoryctes typhlops*	Gadow 1892, Dollo 1899, Bensley 1903, Szalay 1982, 1993, 1994
Genus *Micoureus*	Bensley 1901

### Characterizing Syndactyly

This study approaches marsupial syndactyly from several angles. First, we test the notion that digits II and III evolve as a distinct unit within the syndactylous foot. This is done through assessment of inter-digit integration patterns of morphometric measurements across the diversity of syndactylous marsupials, using a comparison of variance/covariance and correlation matrices. A morphometric approach is also taken to explore the impact of marsupial syndactyly on pedal diversity and functional adaptation. In addition, comparative analysis of ossification sequence in the digits [44-46] is employed to assess the proposition that syndactyly arises through heterochronic change in pedal digit development [13]. Lastly, a review and acquisition of new broad-scale comparative data on external didelphimorph pedal digit morphology, as well as marsupial tarsal articulations, are employed for empirical qualification of previous hypotheses regarding incipient syndactyly and the influence of syndactyly on tarsal morphology.

Using this combination of morphometric, external morphological, and ontogenetic approaches, we address issues regarding the nature, origins, and evolutionary implications of marsupial syndactyly: What are the evolutionary characteristics of digit relationships in syndactylous marsupials? Are these patterns related to heterochronic changes in pedal digit development? Does syndactyly impact on pedal diversity and functional adaptation? Does a reassessment of traditionally used indicators of syndactyly result in a better understanding of its evolution? The results are synthesized into an integrated view of marsupial syndactyly as a unique phenomenon in tetrapod autopodial evolution.

## Results

### Correlation matrix comparisons

Morphometric raw data are shown in Additional file 1. Variance/Covariance (v/cv) and correlation matrix correlations are listed in Table 2. Matrix repeatabilities (listed in the diagonals of Table 2) are mostly over 0.9, suggesting low sampling error. One exception are the repeatabilities for v/cv matrices of digits II and III which are considerably lower (this is probably due to size variation, which is greater than in the remaining digits). This is likely an underestimate since the lower repeatabilities for these digits lead to over-adjustment of their raw v/cv matrix correlations (which is very high; 0.96) to a correlation value over 1. The highest matrix correlations reported by both analyses are those between digits II and III. Medium to high correlations are reported for comparisons of digit V with all other digits, while matrices of digits IV compared with digits II and III have the lowest correlations. Variance/covariance matrix correlations are consistently higher that correlation matrix comparisons, except in comparisons between the syndactylous digits. This difference might be due to the scaling effect on the non-size adjusted data resulting from division by the standard deviation in the correlation approach [47].

**Table 2:** Variance/Covariance and correlation matrix correlations between digits. Matrix repeatabilities are on the diagonal of the matrix.

Variance/covariance	II	III	IV	V
	
II	0.78			
III	1.18	0.83		
IV	0.65	0.74	0.98	
V	0.90	0.95	0.97	0.97
**Correlation**				
II	0.99			
III	1.00	0.98		
IV	0.60	0.61	0.91	
V	0.87	0.89	0.81	0.96

**Figure 3:**
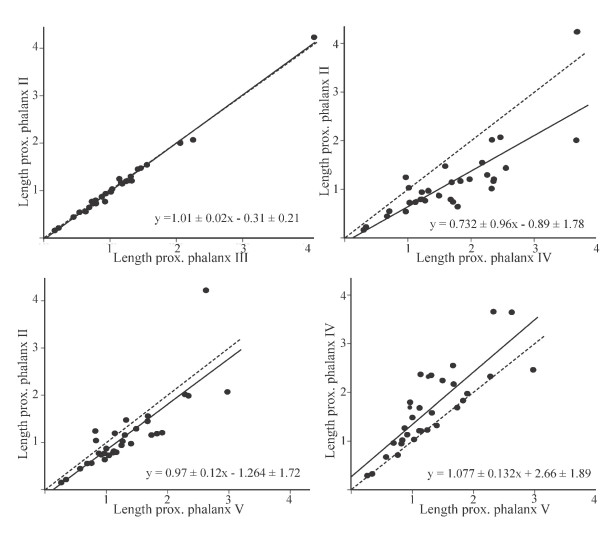
**Raw data regressions**. Representative regression plots of proximal phalangeal length of different digits. Proximal phalanges of digits II and III are virtually isometric with very little error, whereas all others, although sometimes close to isometric, have considerably larger error around the regression line.

The correlations between raw lengths are significantly stronger between phalangeal elements of digits II and III compared to those of digits II or III with digits IV or V (Table 3). Regressions of raw measurements of corresponding digit elements show that this is due to a virtually isometric relationship between digits II and III as evidenced by regression slopes close to 1, with very low regression errors. Same-element regressions between all other digit combinations are considerably different in slope, with larger errors (Table 3; for some examples, see Fig. 3).

**Table 3:** Raw data regression results

	Regression				Correlation comparisons between II/III and others		
	Intercept	*Error*	Slope	*Error*	r	dz	*p*
Metatarsals							
II and III	0.333	*0.924*	0.907	*0.028*	0.986		
II and IV	1.167	*1.451*	0.766	*0.038*	0.965	1.790	0.074
II and V	4.103	*2.421*	0.746	*0.069*	0.892	3.931	0.000
IV and V	2.224	*1.422*	1.029	*0.040*	0.978	0.853	0.394

Pr. Phalanges							
II and III	-0.308	*0.210*	1.014	*0.016*	0.996		
II and IV	-0.886	*1.777*	0.732	*0.096*	0.812	7.395	0.000
II and V	-1.264	*1.722*	0.968	*0.120*	0.828	7.128	0.000
IV and V	2.659	*1.892*	1.077	*0.132*	0.831	7.170	0.000

Int. Phalanges							
II and III	-0.552	*0.352*	1.087	*0.043*	0.977		
II and IV	-1.314	*1.843*	0.729	*0.160*	0.641	5.57	0.000
II and V	-2.208	*1.390*	1.073	*0.155*	0.784	4.41	0.000
IV and V	2.866	*1.212*	0.948	*0.788*	0.788	4.340	0.000

Regression results of raw length regressions between corresponding digit elements. Note that elements of digits II and III are close to identical in length in all species as shown by intercepts near 0 and slopes close to 1 with very little error. This is also reflected in tight correlations between digits II and III, which are nearly all significantly stronger than any other between-digit correlations (the relatively weaker results in metatarsals comparisons are caused by a single high-leverage outlier, *Diprotodon optatum*). r, Pearson correlation coefficient; dz, difference of z-score transformed correlations; *p*, significance.

### Locomotor indices

The Kruskal-Wallis tests of single-digit comparisons suggest highly significant differences between all locomotor types and all predictor ratios (Additional file 2). The post hoc Wilcoxon tests are generally significant between all pairwise group comparisons. The results suggest that arboreal species have longer and more gracile phalanges (see also Figure 2a, b), as demonstrated by higher phalangeal indices, metatarsal slenderness ratios (SRs), distal SRs, and proximal SRs of digits IV and V compared to quadrupedal terrestrial ones. Metatarsal SRs are highest in peramelemorphs and macropodoid species, reflecting their elongate metatarsals (see also Figs. 2c, d).

Kruskal-Wallis tests of locomotor index ratios in digit pairs shows that most between-digit relationships are significantly different between locomotor types. Exceptions are ratios between digits II and III, and all between-metatarsal ratios. The follow-up Wilcoxon test (Additional file 2) is significant for most inter-digit relationships between all three groups, reflecting the markedly different relative digit lengths in species of different locomotor type.

### Digit ossification sequences

**Figure 4:**
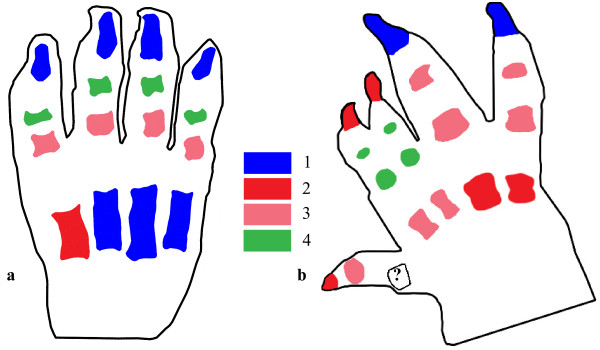
**Pedal digit ossification in syndactylous and non-syndactylous marsupials**. Schematic drawing of ossifications in the foot of a non-syndactylous pouch young Quoll ("native cat") *Dasyurus viverrinus *and syndactylous pygmy possum *Cercartetus concinnus *Elements are coloured according to the sequence in which they ossify, from first (1) to last (4). Not to scale.

**Figure 5:**
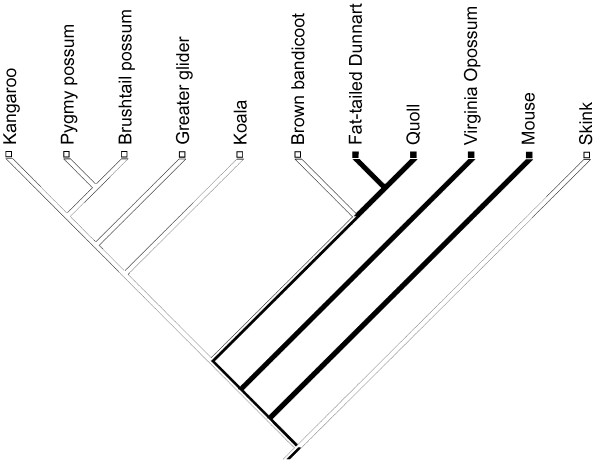
**Event pair mapping**. Phylogeny of the species used for ossification analysis of digit ossifications, with mapping of the example of scores for intermediate digit IV versus proximal digit II. In all syndactylous marsupials, intermediate digit IV ossifies before proximal digit II (white lines), while both ossifications appear simultaneously in all other mammals (solid black line).

Ossification ranks for each species are presented in Additional file 3. In non-syndactylous marsupials, phalangeal elements of one row (e.g. proximal, intermediate) occur simultaneously in digits II-IV (Fig. 4a). In syndactylous marsupials (Fig. 4b), the majority of phalangeal row ossification sequences are disassociated in time: digit IV is earliest to ossify in a row, in most cases simultaneous with or followed by digit V. Then follow ossification of digits II and III (Fig. 4b). Ossification onsets for digit I are slightly more variable, and metatarsals ossify at the same time in all species except for *Cercartetus concinnus *(Fig. 4b). Parsimov analysis of the ossification data, using the consensus of ACCTRAN and DELTRAN optimized apomorphy list inputs, identifies two heterochronic shifts responsible for the rank changes: firstly, an acceleration of intermediate phalanges of digits IV and V with respect to proximal and intermediate phalanges of digits II and III (see Fig. 5 for an example), and secondly, acceleration of distal phalanges IV and V with respect to distal phalanges II and III.

### Tarsal bone anatomy and "incipient syndactyly"

All marsupial species examined, except peramelemorphs, show metatarsal-tarsal articulations that correspond to the plesiomorphic mammalian pattern (Fig. 2a–c). Digits II and III articulate with the mesocuneiform and ectocuneiform bone, respectively, in all marsupials, while in peramelemorphs, digit III articulates perceptibly less with the ectocuneiform. Digit IV is mostly supported by the cuboid in all species except peramelemorphs, where it is supported partially by the ectocuneiform and cuboid bones (Fig. 2d). However, in wombats and a range of macropodoids there is also considerable contact between digit IV and the ectocuneiform (Fig. 2c). Within peramelemorphs, there is some variation as to the degree to which the ectocuneiform is included in support of digit IV. This seems to be related to locomotor mode, as the most extensive contact is in the cursorial bilbies, *Macrotis lagotis *and the extinct *Chaeropus ecaudatus*. Digit V is supported by the cuboid in all species.

**Figure 6:**
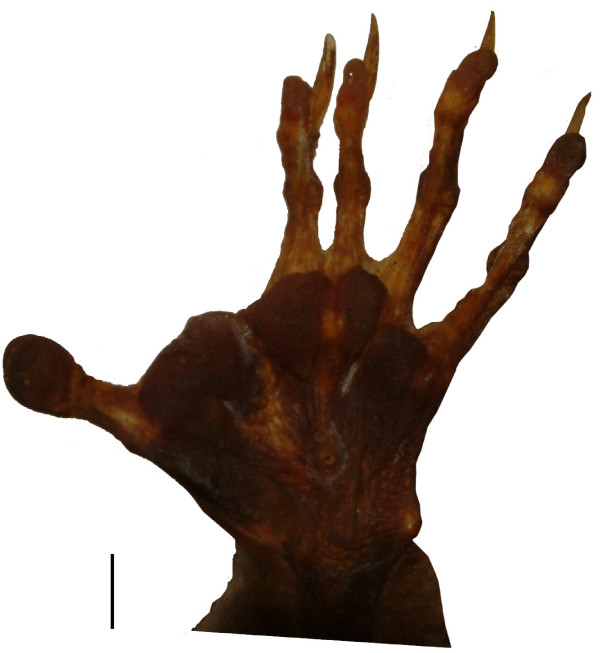
**Foot of *Caluromys derbianus*. **Palmar view of the foot of the didelphimorph *Caluromys derbianus *showing shorter, unwebbed digits II and III; this occurs in some didelphimorphs and has been considered "incipient syndactyly". However, these digit proportions are common in arboreal mammals and do not seem equivalent to syndactyly. Scale bar = 1 cm

Species for which claims of "incipient syndactyly" were found in the literature are listed in Table 1. Visual investigation of study skins of didelphimorph marsupials show that in most species, all middle digits (digits II-IV) are highly similar. In some didelphimorphs, digits II and III are more gracile than digits IV and V, and more similar to each other (for an example, see Fig. 6), but never as distinctly so as in syndactylous marsupials. In no case are digits II and III fully webbed, except in the fully webbed feet of *Chironectes minimus *and in the foot of *Notoryctes typhlops*, whose digits I-IV are webbed.

## Discussion

The origins and evolution of marsupial syndactyly have been debated for more than 150 years, although little observational information has been collected. It may be that the distinctive overall anatomy of syndactyly resulted in the perception that it is distinctive enough not to require further research. However, the results of this study indicate that marsupial syndactyly has a unique evolutionary and developmental background which has previously been largely ignored. A synthesis of results from this study takes the implications of this unique phenomenon beyond its traditional role as a purely phylogenetic problem.

### Unique integration in the syndactylous foot

The near-identity of correlation matrices of digits II and III, combined with strict raw measurement isometry of their digit elements across species, suggest that the syndactylous digits are highly constrained with respect to each other across the diversity of syndactylous species. This is congruent with the fact that the phalangeal skeleton of the syndactylous digits is visually undistinguishable. This is reminiscent of a definition of identical digits by Tabin [[48], p. 290]: " Empirically, a digit is considered to have the same identity as a second digit 'x' if, when examined in isolation by a morphologist, the first digit would be labeled as being a digit 'x'". Such a condition has few counterparts in extant tetrapods. Among cases of marsupial-like syndactyly reported in placentals, only that in digit III and IV in three closely related species of otter shrew (*Potamogale velox *and two species of *Micropotamogale*) is directly comparable [49]; note that the reportedly syndactylous siamang gibbon *Symphalangus syndactylus *has digits of different length [50]. To our knowledge, conditions resembling marsupial syndactyly are also unknown in extant amphibians or sauropsids [51]. It could be argued that digits III and IV of hoofed placentals (artiodactyla and perissodactyla) could be comparable in their similarity to marsupial syndactyly. However, autopodia of hoofed placentals follow a well-documented pattern of increased central digit emphasis in fore-and hindlimbs which is restricted to cursorial mammals [32,52,53]. Marsupial syndactyly is not comparable since it is restricted to the foot and morphologically and functionally diverse, and does not correspond to any obvious locomotor adaptation.

### Syndactyly does not constrain functional adaptation of the foot

Autopodial anatomy in tetrapods is strongly tied to locomotor function [54-56], and this is reflected in greater variation in autopodial anatomy compared to more proximally situated limb elements [57,58]. Extensive locomotor adaptation within pedal digits is evident from the matrix comparisons involving digits II or III versus IV and V. Digit and phalangeal proportions correspond with those generally predicted for mammalian autopodia [7,55,56,59,60]: arboreal species have higher phalangeal indices and phalangeal slenderness ratios, while species with exclusively terrestrial locomotion have shorter phalanges and more sturdy phalangeal proportions. Between-digit ratio differences also reveal considerable differentiation of digits with respect to each other (except II and III) according to locomotion. Although not morphometrically assessed, digit I also follows widely recognized patterns of functional adaptation [52]: it is strongly developed and opposable in the arboreal possums and koalas, and reduced or absent in the hopping or bounding kangaroos and bandicoots, which is consistent with patterns of marginal digit loss observed in cursorial, bounding and hopping mammals [37,52,56].

The fact that syndactylous digit proportions differ significantly between locomotor groups supports the hypothesis that they evolve as a unit subject to selection pressures. This complements the results of dissection studies which show that the muscles operating the syndactylous digits are as well developed and functional as in non-syndactylous species [summarized in [3,33]]. In this respect, length similarity of syndactylous phalangeal elements may hold an important implication for their mobility: because the digits are tightly appressed under a common skin sheath and bound by connective tissue [3], their capacity to flex and extend depends on alignment of the joints along a plane that allows them to move together.

It is notable that proportions and morphology of all pedal digits in macropodoids (kangaroos) and peramelemorphs are highly similar (see Figure 2c, d). This includes an enlarged digit IV as the main axis of the foot, a slightly smaller digit V, very thin and gracile digits II and III, and reduced or absent digit I. This convergence is interesting because emphasis of central digits often coincides with evolutionary trends in placentals towards cursoriality or bounding locomotion [37,61]. Tyndale-Biscoe [62] suggested that syndactyly arose as an adaptation to cursoriality or bounding in Peramelemorpha and Diprotodontia. This is unlikely if it is true that the ancestors of both clades were arboreal [6,8,16,25,28,36]. Conversely, however, the functional loss of a digit in syndactylous feet may have favoured the evolution of the bounding or half-bounding habit in both clades.

### Syndactyly may not be a functional-adaptive trait

The few studies that have been concerned with the origins of syndactyly have invoked functional-adaptive scenarios for their appearance, either as grooming organs [3,11,18,31] or as an adaptation to arboreality [13,28,29]. However, marsupial syndactyly does not resemble any autopodial adaptations ever evolved in mammalian autopodia. The characteristics of syndactyly are unique among terrestrial mammalian orders (except for three species of otter shrews). The coinciding heterochrony and integration in syndactylous digits also cannot be explained by simple processes of locomotor adaptation, particularly because these patterns are conservative across the considerable diversity of locomotor types in Diprotodontia. Rather, syndactyly may represent a constraint that the foot is adapted to. Under this scenario, digits IV and V, which are always well developed, compensate for the "unification" of the two syndactylous digits. An interesting further implication of viewing syndactyly as a constraint would be a new view of the perceived "reduction" of the syndactylous digits, which has been taken to be a hallmark of syndactyly [3,13,21,28]. If digits II and III were constrained to evolve as a single digit and functional unit, it would be expected that the most advantageous dimensions of this complex would be that of a single digit, which is indeed reflected in the dimensions of the syndactylous complex [13]. This would also explain why the much-noted "reduction" is not a general feature of marsupial syndactylous digits. For example, in most arboreal species the syndactylous digits are well developed [[3]; see also Fig. 2a, b]. In fact, the syndactylous digit complex in wombats is longer, although not wider, than digits IV and V (pers. obs.), which is congruent with their digging habit [56,61]. As such, there is evidence to suggest that the "reduction" of digits is not an intrinsic property of syndactyly, but may be it's consequence.

### Character distribution of syndactyly

The question as to whether marsupial syndactyly arose once or twice has been discussed extensively, mainly because convergent origins of this perceivedly complex character have been deemed unlikely [16]. A sister group relationship between Peramelemorpha and Diprotodontia, and as such a single origin for syndactyly in these two clades, can be ruled out based on the latest phylogenies. However, it has been suggested that syndactyly may be in fact homoplastic within a larger marsupial clade [21,23,27]. Support for this notion comes from possible tracks of a syndactylous marsupial from the Cretaceous of British Colombia [63] and, tentatively, from the Late Cretaceous of Western Colorado [64]. Moreover, claims of "incipient syndactyly" in some South American Didelphimorphia and the marsupial mole *Notoryctes typhlops *have been considered evidence of an older origin of syndactyly. Extensive connection of digits II and III by skin was only observed in the fully webbed feet of the semiaquatic Yapok *Chironectes minimus *and the mostly webbed feet of *Notoryctes typhlops*, but digits II and III and their elements do not resemble each other and are not closely appressed [3]. The sporadic occurrence of digits II and III which are shorter than digit IV in some didelphimorphs is not unusual for mammals. It is neither as highly conserved as syndactyly in Diprotodontia and Peramelemorpha, nor does it resemble it to a great degree. It is possible that the shorter length of digits II and III in these species is due to their mostly arboreal or scansorial habit [49], which commonly coincides with short medial digits [7,55,65,66]. The results of this study therefore suggest that syndactyly is convergent in Diprotodontia and Peramelemorpha, and is not plesiomorphic for marsupials.

### Scenarios for the origins of syndactyly

Given the extensive similarities of peramelemorph and diprotodontian syndactyly, the convergent evolution of this trait in the two clades appears unlikely to have occurred at random. It is possible that convergence of syndactyly constitutes a case of parallelism which develops through similar or identical developmental pathways [1]. This would suggest that that there is a greater likelihood of syndactyly evolving in peramelemorphs and diprotodontians due to shared ancestral developmental patterns. Occurrence of full syndactyly in early marsupials and/or metatherians, as has been suggested based on Cretaceous tracks [63,64], is also conceivable under this scenario.

How syndactyly could establish itself twice, and the nature of the developmental changes involved, are difficult to establish in the absence of a functional-adaptive scenario. However, the results of this study provide circumstantial evidence that allow the position of hypotheses for further testing. Integration and isometry of digits II and III can be explained in two ways; firstly, as an adaptation to a bounding of digits by skin, and secondly, as the result of a change in ontogenetic patterns that "synchronized" the morphology of the syndactylous digits. Under the first scenario, syndactyly would have established itself as a malformation, possibly during a time when the ancestral populations were undergoing a bottleneck event [13]; the length isometry of the digits could have arisen because this allowed the digits to function as a unit (see above). However, this includes the unparsimonious scenario of a malformation establishing itself in two unrelated marsupial populations, with subsequent highly similar morphological outcomes. Also, if syndactyly were just a skin fusion of the digits, this would not explain the ossification heterochrony that coincides with syndactyly in Diprotodontia and Peramelemorpha.

The second scenario, positing an ontogenetic pattern change, may be more parsimonious and is favoured by some of the evidence from this study. If the morphometric integration of digit elements is intrinsic to syndactyly through an ontogenetic constraint, the establishment of syndactyly as a non-adaptive trait is less improbable because, as discussed above, it effectively retains the capacity of digit flexion and extension. As such, syndactyly would only represent the loss of a single digit, rather than the incapacitation of both digits as in soft-tissue syndactyly of the human hand [67]. Digit loss is common in mammals, although marginal digits are usually affected [32,36,37,52]. With functional syndactylous digits, the net loss of a single digit may have had a mild impact, compared to a loss of flexing capacity, as is the case in syndactylous human hands. This may have aided the spread of marsupial syndactyly through the ancestral populations.

Phenotypes resembling marsupial syndactyly in that the digits display identical morphologies have existed in some of the earliest tetrapods such as *Acanthostega*; this similarity has been ascribed to identical patterns of morphogen expression in the digits [48]. Identical digits have also been created through biochemical manipulation of developing autopodia of mice, chicks, and frogs [68-71]. These experiments largely involve alteration in the expression or concentration of morphogens [mostly bmp family transcription factors; [68,69,72,73]]. It is notable that many of these are intimately associated with retention of the inter-digital membrane as it occurs in syndactylous marsupials. As ossification heterochrony and integration of digits II and III hint at ontogenetic changes in these two digits, it should be tested whether the switch to syndactyly involves a naturally occuring change in one of these pathways. Should this be the case, it would be predicted that the ancestral phenotype of syndactyly arose rapidly and was largely similar to that of the more plesiomorphic syndactylous marsupials today. This would provide an avenue for rapid origin of fully integrated, and as such fully functional, syndactylous digits. It would also explain the lack of intermediates of syndactylous feet in extant or extinct marsupials and the heterochrony observed in this study. A similar scenario has been argued by Sears [40] with respect to the origin of bat wings, explaining the conservative size relationship of digits across bat species, as well as the lack of fossil intermediates of bat wings, with a localized change in bmp expression patterns.

### Tarsal-metatarsal articulations are not impacted by syndactyly

The generalized pattern of tarsal-metatarsal articulations throughout marsupials is that digit I articulates with the ectocuneiform, digit II with the mesocuneiform, digit III with the ectocuneiform, and digits IV and V articulate with the cuboid bone. This pattern is highly conserved among marsupials and is already present in the oldest metatherian tarsus known to date, belonging to *Sinodelphys szalayi *[74]. Woodburne and Case [27] argued that differences of tarsal/metatarsal arrangement in Diprotodontia and Peramelemorpha suggest convergent evolution of syndactyly in these clades. However, the results of this study disagree with Woodburne and Case's [27] reports in several points. Their claim that the endocuneiform is lost in macropodoids cannot be confirmed [see also [25]], and no evidence was found to support a "lateral dislocation" of peramelemorph metatarsals with respect to tarsals. Indeed, no difference in articulation patterns of peramelemorphs compared to other marsupials was noted, other than that pertaining to digit IV. Given the conservatism of tarsal anatomy with respect to metatarsals II and III across all marsupials, it seems that syndactyly is a phenomenon exclusively confined to digit morphology and has no impact on tarsal morphology. The peramelemorph tarsal arrangement also appears in hoofed placentals, where it is considered to represent an adaptation to the emphasis on the central digits [15]. It is noteworthy that kangaroos, whose plesiomorphic locomotion patterns resemble those of peramelemorphs [75], also show a tendency towards increased contact between metatarsal IV and the ectocuneiform. This suggests that the extensive contact between metatarsal IV and the ectocuneiform in peramelemorphs is related to a stabilizing re-arrangement, rather than a result of syndactyly.

### Marsupial and human syndactyly

Marsupial syndactyly derives its name from a congenital malformation in humans, and has been treated as directly comparable [13,23,42,50,62]. Human syndactyly is an overarching term for a diverse congenital condition which always involves incomplete digit separation during early development due to a lack of inter-digit membrane cell death, or through synostoses [76]. Marsupial syndactyly only corresponds to a mild subtype of syndactyly termed "zygodactyly" [77,78]. However, in its worst form human syndactyly can involve synostoses of the majority of digit elements [76]. Contrary to a popular notion in the field of marsupial systematics [21,23,42,62] the genetic background of syndactyly is complicated and only known for a few of the over 50 types [76,77]. It is notable that even the mildest forms of syndactyly in the hand lead to incapacitation of grasping capability of the affected digits and require surgical treatment, although they are rarely separated in the foot [67]. Human syndactyly is only partly heritable, and within families where syndactyly is passed on, it manifests variably in terms of severity, the limb affected, and number of digits involved [77]. The present results reveal that marsupial syndactyly is a highly specific phenotype that does not compare to human syndactyly more than superficially because marsupial syndactyly is entirely heritable and highly conservative in its manifestation. As such, the term "syndactyly" is slightly unfortunate for the unique condition found in marsupials, and has in the past lead to the uncritical assumption that the two conditions are directly comparable. It may be desirable to clearly distinguish between the two conditions by consistently referring to marsupial syndactyly as such, or introducing a new term (for example, "homodactyly" may be appropriate given the similarity of digits II and III) altogether.

## Conclusion

The diverse methods employed in this study have provided novel insights on the phenomenon of marsupial syndactyly. The results demonstrate that the syndactylous digits of marsupials evolve as a unit which is subject to functional adaptation like the remaining digits of the foot. The strong integration between the two digits may be due to a change in early developmental patterning, as the ossification heterochrony between syndactylous compared to non-syndactylous species suggests. No evidence was found for incipient syndactyly in other marsupials, but the ossification similarity of pedal digits in Diprotodontia and Peramelemorpha suggest an underlying developmental parallelism. We argue that locomotor adaptation is not likely to be the cause for syndactyly; based on our results, we suggest a scenario in which syndactyly arose as a single change in digit ontogeny that amounted to the loss of a single digit, rather than incapacitation of both digits through skin webbing. We also show that syndactyly is restricted to Peramelemorpha and Diprotodontia, and has no influence on tarsal bone arrangement in either clade. Together, the results change the relevance of marsupial syndactyly from being an enigmatic phylogenetic character within marsupials to a rare transformation of digit morphology whose molecular background has the potential to provide important insights into the patterns of digit evolution.

## Methods

### Morphometric data collection

Articulated pedal skeletons from specimens of 32 syndactylous marsupial species were measured. Species were selected to include the full range of pedal diversity in syndactylous marsupials. Raw measurements and accession numbers can be found in Additional files 1 and 4; for terminology, refer to Figure 2a. Measurements of 11 phalangeroid and petauroid possums, 11 kangaroos, 7 vombatiforms (four extinct), and 4 peramelemorphs were taken using digital calipers (specified accuracy ± 0.02 mm). In most extant species, two adult specimens per species were measured and measurements were averaged following Christiansen [79]; all individual measurements were taken twice and averaged. Measurements comprised length and mid-shaft width of metatarsals, proximal and intermediate phalanges II-V (a total of 6 measurements per digit). Measurements for digit I were not taken since this digit is reduced or lost in kangaroos and bandicoots.

### Matrix comparisons and assessment of raw measurements of non-size adjusted data

To assess inter-digit integration patterns across the diversity of syndactylous marsupials, a variance/covariance and correlation matrix comparison approach was employed. Matrix comparisons are suited for this problem because they can quantify integration patterns that may constrain morphological variability of the trait components with respect to each other [58,80-83]. Natural logarithm-transformed morphometric measurements were investigated. The measurements were not otherwise adjusted by size because relative size variation is an important factor in differences between the syndactylous and non-syndactylous digits. Correlation and variance/covariance matrices of digit measurements were computed for each digit, resulting in 6 × 6 matrices. Correlation matrices were compared using a Mantel's test [84] implemented by the freeware Microsoft Excel-addin PopTools [85], which compares the matrices and provides a non-parametric estimate for the significance of the correlation. Variance-covariance matrices were compared using the random skewers method [82,86], run through a Monte-Carlo simulation implemented by PopTools [58]. Matrix repeatabilities were also computed as a measure of sampling error following Cheverud [80] and Marroig and Cheverud [82] using the Monte-Carlo simulation routine in PopTools.

Matrix correlations provide an estimate of correlated evolution between the traits studied, but they do not provide information on absolute size relationships. In other words, two anatomical complexes can have highly similar matrices but be of greatly differing absolute sizes. As noted above, size relationships between digits are a crucial component in this study because syndactyly manifests itself in unusually "synchronized" – looking size relationships between digits II and III. To address this, raw length measurements were plotted for visual assessment and their correlations were compared using Fisher's z test [87].

### Locomotor indices – proportional differences

Morphometric correlates of locomotion of non-syndactylous autopodial anatomy are well understood [7,52], so that it is possible test whether the syndactylous foot is subject to generally recognized patterns of locomotor adaptation. The species measured were divided into three locomotor groups: hopping and bounding species (kangaroos/rat kangaroos and bandicoots), arboreal species (phalangeroid and petauroid possums), and terrestrial plantigrade species (vombatiforms). Based on the morphometric measurements, differences in morphometric locomotor predictor indices were computed. Using ratio-based locomotor indices is a convenient way of focusing on that part of the variation which is explained exclusively by proportional, rather than size-related, differences. The phalangeal index [proximal and intermediate phalangeal length as a percentage of metatarsal length; [59,88]] and metatarsal, proximal phalangeal, and intermediate slenderness ratios [SRs; element length*100/element width; [7]] were employed because they are known to be reliable proxies of locomotor habit. Locomotor index comparisons were conducted on two levels: Firstly, measurements in single digits were compared between locomotor groups. Secondly, ratios between locomotor indices of digit pairs were compared to capture some of the disparities in between-digit proportions in species of different locomotor types.

Because of disparate and relatively small sample sizes in each locomotor group, the non-parametric Kruskal-Wallis test was performed to assess whether significant differences existed within the three locomotor groups. Indices for which significant results were found were also investigated using post-hoc Wilcoxon signed rank sum tests to assess whether all three locomotor types are distinguished using the predictor indices.

### Digit ossification sequences

To investigate pedal digit ontogeny in syndactylous marsupials, ossification sequences of pedal digit elements were recorded for ontogenetic series of 5 diprotodontian species, the peramelemorph *Isoodon macroura*, 2 dasyurids, the ameridelphian *Didelphis virginiana*, the placental mouse *Mus musculus*, and the skincid lizard *Hemiergis peronii *as an outgroup. Museum accession numbers are listed in Additional file 4. Sequence data were collected from the literature for *Hemiergis peronii *[89], *Mus musculus *[54], *Sminthopsis macroura *[90], and *Didelphis virginiana *[91,92]. Pouch young from the personal collection of VW were clear stained according to Dingerkus and Uhler [93], modified by Prochel [94]. The remaining specimens were investigated by acquiring X-ray images (using a SkyScan 1172 desktop micro-computer tomography scanner) of the specimen's lower half of the body, taken at 30° intervals during a 360° rotation to allow a multi-angle view of the foot.

The ossification onset of metacarpals and phalangeal elements in all digits was recorded. For each species, ranks were given to ossifications in the order in which they occur, and compared across species. Sequence heterochrony in the appearance of ossification sequences was also summarized by coding the sequences into event pairs [46,95] and analysing these using the Parsimov program package [96]. The consensus of ACCTRAN and DELTRAN optimized analyses is presented here. This means that ambiguous transformations are not considered, which results in a conservative but more reliable estimate [96]. Because the sample for peramelemorphs contained only one poorly resolved species, Parsimov was run without it.

### Tarsal-metatarsal articulations and "incipient syndactyly"

The tarsal-metatarsal joint was investigated in 23 species from 5 marsupial orders. Accession numbers can be found in Additional file 4; for terminology, refer to Figure 2a. When possible, several individuals per species were investigated. The tarsal/metatarsal articulations were listed for each species.

A literature search for reports of "incipient syndactyly" was conducted, and species for which reports exist were re-investigated (Table 1). This was done using study skins whose pedal skeleton was left in the skin, and articulated pedal skeletons (for accession numbers, see Additional file 4).

## Authors' contributions

VW wrote the manuscript, collected and analysed the morphometric data and most ossification data except those obtained from clearing and staining. MN conducted the clearing and staining on specimens provided by VW and collected and analysed data on tarsal morphology and incipient syndactyly. MN also reviewed the literature on the historical, phylogenetic, and molecular context of syndactyly and contributed to the drafting of the manuscript. Both authors read and approved the final manuscript.

## Supplementary Material

Additional file 1 Raw morphometric data. Raw morphometric data Click here for file

Additional file 2 Kruskal-Wallis and Wilcoxon rank-sum test results for differences in locomotor indices. Rank-sum test results for differences in locomotor indices. Click here for file

Additional file 3 Ossification data. Ranks of timing, from earliest to latest, for the species considered in the analysis of pedal digit ontogeny. Click here for file

Additional file 4 Accession numbers. Accession numbers or sources for investigations related to ossification sequence, tarsal/metatarsal, and "incipient syndactyly". Click here for file
