# Supplementary material for: Integration, heterochrony, and adaptation in pedal digits of syndactylous marsupials
Source: BMC Evol Biol. 2008 May 25;8:160. doi: 10.1186/1471-2148-8-160 (PMC2430710; doi:10.1186/1471-2148-8-160)
Supplement: Additional file 2 — Kruskal-Wallis and Wilcoxon rank-sum test results for differences in locomotor indices. Rank-sum test results for differences in locomotor indices. [file 1471-2148-8-160-S2.doc]

**Appendix 2:** Kruskal-Wallis and Wilcoxon rank-sum test results for differences in locomotor indices.

|  | Kruskal-Wallis test | | Non-significant comparisons in the Wilcoxon rank-sum tests^1^ |
| --- | --- | --- | --- |
|  | Chi Square | *p* |  |
| **Single indices** |  |  |  |
| Phal. index II | 22.571 | 0.000*** | plantigrade vs. arboreal |
| Phal. index III | 23.458 | 0.000*** |  |
| Phal. index IV | 21.740 | 0.000*** | plantigrade vs. hopping |
| Phal. index V | 23.395 | 0.000*** |  |
| MSR II | 23.358 | 0.000*** |  |
| MSR III | 25.161 | 0.000*** |  |
| MSR IV | 16.289 | 0.000*** |  |
| MSR V | 20.872 | 0.000*** |  |
| PSR II | 19.165 | 0.000*** |  |
| PSR III | 17.849 | 0.000*** |  |
| PSR IV | 14.400 | 0.000*** | Hopping vs. arboreal |
| PSR V | 16.309 | 0.000*** |  |
| ISR II | 9.382 | 0.009** |  |
| ISR III | 15.603 | 0.000*** | plantigrade vs. hopping |
| ISR IV | 15.990 | 0.000*** |  |
| ISR V | 20.788 | 0.000*** |  |
| **Between-digit relationships** |  |  |  |
| Length II/III | 3.294 | 0.192 |  |
| Length III/IV | 19.040 | 0.000*** |  |
| Length III/V | 5.591 | 0.061 |  |
| Length IV/V | 16.843 | 0.000*** | plantigrade vs. arb., hopping |
| Phal. index II/III | 1.767 | 0.413 |  |
| Phal. index III/IV | 24.788 | 0.000*** |  |
| Phal. index III/V | 10.662 | 0.000*** |  |
| Phal. index IV/V | 23.141 | 0.000*** |  |
| MSR II/III | 2.469 | 0.291 |  |
| MSR IV/III | 22.192 | 0.000*** |  |
| MSR III/V | 7.430 | 0.024* |  |
| MSR IV/V | 24.656 | 0.000*** |  |
| PSR II/III | 0.008 | 0.996 |  |
| PSR III/IV | 20.290 | 0.000*** |  |
| PSR III/V | 16.813 | 0.000*** |  |
| PSR IV/V | 9.074 | 0.011* |  |
| ISR II/II | 3.511 | 0.172 |  |
| ISR IV/III | 12.502 | 0.002** |  |
| ISR III/V | 10.332 | 0.006** |  |
| ISR IV/V | 18.830 | 0.000*** |  |

Abbreviations: ISR, Intermediate phalanx slenderness index; MSR, Metatarsal slenderness index; Phal. index, Phalangeal index; PSR, Proximal phalanx slenderness index. ^1^ only comparisons for which the Kruskal-Wallis test was significant were examined with the Wilcoxon rank-sum test.
