# Supplementary material for: Integration, heterochrony, and adaptation in pedal digits of syndactylous marsupials
Source: BMC Evol Biol. 2008 May 25;8:160. doi: 10.1186/1471-2148-8-160 (PMC2430710; doi:10.1186/1471-2148-8-160)
Supplement: Additional file 3 — Ossification data. Ranks of timing, from earliest to latest, for the species considered in the analysis of pedal digit ontogeny. [file 1471-2148-8-160-S3.doc]

**Appendix 3:** Ranks of timing, from earliest to latest, for the species considered in the analysis of pedal digit ontogeny

| *Hemiergis peronii* | | *Mus musculus* | | *Didelphis virginiana* | | *Dasyurus viverrinus* | | *Sminthopsis macroura* | | *Isoodon obesulus* | | *Macropus eugenii* | | *Cercartetus concinnus* | | *Phascolarct. cinereus* | | *Petaurus breviceps* | | *Trichosurus vulpecula* | |
| --- | --- | --- | --- | --- | --- | --- | --- | --- | --- | --- | --- | --- | --- | --- | --- | --- | --- | --- | --- | --- | --- |
| n=8 |  | n=13 |  |  |  | n=11 |  | n=11 |  | n=7 |  | n=11 |  | n=25 |  | n=5 |  | n=29 |  | n=13 |  |
| M I | - | M II | 1 | D II | 1 | MIT | - | M I | 0 | MIT | - | MIT | - | M I | ? | D IV | 1 | D IV | 1 | D IV | 1 |
| P I | - | M III | 1 | D III | 1 | P I | - | P I | 0 | P I | - | P I | - | D IV | 1 | D V | 1 | D V | 1 | D V | 2 |
| D I | - | M IV | 1 | D IV | 1 | D I | - | D I | 0 | D I | - | D I | - | D V | 1 | M I | 2 | D II | 2 | D I | 3 |
| M II | 1 | M I | 2 | D V | 1 | D II | 1 | M II | 1 | M II | 1 | D IV | 1 | D I | 2 | P I | 2 | D III | 2 | M II | 3 |
| M III | 1 | D I | 2 | M II | 2 | M III | 1 | M III | 1 | D II | 1 | M II | 2 | D II | 2 | D I | 2 | M I | 3 | M III | 3 |
| P III | 1 | D II | 2 | M III | 2 | D III | 1 | M IV | 1 | M III | 1 | M III | 2 | D III | 2 | M II | 2 | P I | 3 | D II | 3 |
| M IV | 1 | D III | 2 | M IV | 2 | M IV | 1 | M V | 1 | D III | 1 | M IV | 2 | M IV | 2 | P II | 2 | D I | 3 | D III | 3 |
| P IV | 1 | D IV | 2 | P II | 2 | D IV | 1 | P II | 2 | M IV | 1 | M V | 2 | M V | 2 | D II | 2 | M II | 3 | M IV | 3 |
| I IV | 1 | M V | 2 | P III | 2 | M V | 1 | D II | 2 | D IV | 1 | D V | 2 | M II | 3 | M III | 2 | P II | 3 | M V | 3 |
| P II | 2 | D V | 2 | P IV | 2 | D V | 1 | P III | 2 | M V | 1 | D II | 3 | M III | 3 | P III | 2 | M III | 3 | M I | 4 |
| I II | 2 | P I | 3 | P I | 3 | M II | 2 | D III | 2 | D V | 1 | D III | 3 | I IV | 3 | D III | 2 | P III | 3 | P I | 4 |
| IIII | 2 | P II | 3 | I II | 3 | P II | 3 | P IV | 2 | P IV | 2 | I IV | 5 | P IV | 3 | M IV | 2 | M IV | 3 | P II | 4 |
| D III | 2 | P III | 3 | IIII | 3 | P III | 3 | D IV | 2 | P II | 3 | P IV | 4 | P V | 3 | P IV | 2 | P IV | 3 | P III | 4 |
| D IV | 2 | P IV | 3 | I IV | 3 | P IV | 3 | P V | 2 | P III | 3 | P V | 5 | I V | 3 | I IV | 2 | I IV | 3 | P IV | 4 |
| M V | 2 | P V | 3 | M V | 3 | P V | 3 | D V | 2 | I IV | 3 | P II | 6 | P I | 3 | M V | 2 | M V | 3 | I IV | 4 |
| P V | 2 | I II | 4 | P V | 3 | I II | 4 | I II | 3 | P V | 3 | P III | 6 | P II | 4 | P V | 2 | P V | 3 | P V | 4 |
| I V | 2 | IIII | 4 | I V | 3 | IIII | 4 | IIII | 3 | I II | 4 | I II | 7 | I II | 4 | I V | 2 | I V | 3 | I V | 4 |
| D II | 3 | I IV | 4 | M I | 4 | I IV | 4 | I IV | 3 | IIII | 4 | IIII | 7 | P III | 4 | I II | 3 | I II | 4 | I II | 5 |
| D V | 3 | I V | 4 | D I | ? | I V | 4 | I V | 3 | I V | 4 | I V | 7 | IIII | 4 | IIII | 3 | IIII | 4 | IIII | 5 |

­Abbreviations: n, number of specimens; D, Distal phalanx; I, intermediate phalanx; M, metatarsal; P, proximal phalanx. Roman numerals refer to digit ray numbers.
