# Supplementary material for: Integration, heterochrony, and adaptation in pedal digits of syndactylous marsupials
Source: BMC Evol Biol. 2008 May 25;8:160. doi: 10.1186/1471-2148-8-160 (PMC2430710; doi:10.1186/1471-2148-8-160)
Supplement: Additional file 4 — Accession numbers. Accession numbers or sources for investigations related to ossification sequence, tarsal/metatarsal, and "incipient syndactyly". [file 1471-2148-8-160-S4.doc]

**Appendix 4:** Accession numbers or sources for investigations related to ossification sequence, tarsal/metatarsal, and "incipient syndactyly". Abbreviations: AUM, Australian Museum; BNHM, British Natural History Museum, London; MQU, Macquarie University donation, MV, Victoria Museum, Melbourne; SAM, South Australian Museum

**Ossification sequences:**

*Dasyurus viverrinus*: AUM 2119, 7057, 7803, 2206, 31115, 3112, 31125, 31772, 3764, 744, 1864

*Isoodon macrourus*: AUM 26198, 32987, 37135, 37139, 37140 and 2 specimens donated by the Adelaide Museum Anatomy department in the personal collection of VW

*Petaurus breviceps:* AUM 25387, 25817, 25912, 27760, 29158, 29165, 29168, 29260, 31133, 31137, 33312, 5303, 5375, 6197, 7233, 24149, 33665, 34664, 34824, 35576, 35583, 36417

*Cercartetus concinnus:* SAM 4046, 18019, 15982, 127450, 22776 ,11640, 13012, 16275, 20687, 16938, 16040, 14411, 4067, 22912, 15907, 22939, 22921, 5665; AUM 26252, 26254, 26253, 31132, 33131, 26257, 26255

*Macropus eugenii*: MQU Coll. No. 6380464, 2044, 8515, 8589, 61F40, 61FC70CT, 62137, 637EAIT, 6380Y, 63BDEBOT, XIAO

*Phascolarctos cinereus:* 5 specimens donated by the Adelaide Museum Anatomy department in the personal collection of VW

*Trichosurus vulpecula:* AUM 3097, 3098, 31153, 33309, 37060, 5013, 5321, 2373, 5 specimens donated by the Adelaide Museum Anatomy department in the personal collection of VW

**Tarsal/metatarsal articulations, all from BNHM:**

*Pseudocheirus occidentalis*: 99.4.21.1

*Petauroides volans*: 48.9.6.1

*Petaurus breviceps*: 245.A

*Vombatus ursinus*: 1950.3.27.2

*Lasiorhinus latifrons*:77.F.13.1

*Phascolarctos cinereus*: 75.18.28; 64.5.7.3

*Dendrolagus inustus*: 1936.2.24.1

*Trichosurus vulpecula*: 53.8.17.2

*Phalanger gymnotis*: 1937.7.16.1

*Phalanger maculatus*: 1983.311

*Macropus ruficollis*: 1948.5.10.5; 1948.5.10.4

*Macropus robustus*: 74.465

*Dorcopsis luctuosa*: 75.4.16.1

*Macrotis lagotis*: 1948.5.10.6; 75.18.17

*Perameles bougainville*: 70.80.30.1

*Dasycerus cristicauda*: 75.1818

*Dasyurus maculatus*: 1936.8.2.91

*Dasyurus hallucatus*: 1966.4.4.2

*Sarcophilus harrisi*: 69.8.11.2

*Nototyctes tyhlops*: 69.8.15.8

*Chironectes minimus*: 849.D; 66.2.15.1

*Caluromys derbianus*: 1962.5.2.3

*Didelphis marsupialis*: 1949.18.2

**"Incipient Syndactyly", all from BNHM:**

*Philander venezuelae*: 11.5.25.172; 11.5.25.174

*Philander trinitatis*: 35.210.2; 97.6.7.21

*Philander laniger guayanus*: 80.5.6.88; 15.7.12.35

*Philander philander*: 47.9.21.3; 23.8.10.34

*Lutreolina crassicaudata*: 25.5112

*Metachirus nudicaudatus*: 24.2.11.27

*Marmosa cinerea*: 14.1.27.8; 2.11.7.17

*Marmosa chloe*: 7.6.20.18

*Marmosa klegesi*: 1.6.4135

*Marmosa noctivaga*: 28.7.21.112; 27.11.1.256

*Marmosa pusilla*: 4.1.5.46; 5.8.1.8

*Marmosa incana*: 3.9.5.145; 3.9.5.140

*Lestodelphis halli*: 28.12.11.206; 28.12.11.205

*Caluromys derbianus*: 13.8.10.14
